# TLR2 activation potentiates P-glycoprotein-mediated methotrexate efflux and enhances cytotoxicity of human NK cells against acute lymphoblastic leukemia

**DOI:** 10.3389/fimmu.2026.1757205

**Published:** 2026-04-15

**Authors:** Pablo Álvarez-Carrasco, Paola Basurto-Olvera, Elva Jiménez-Hernández, Aurora Medina-Sanson, Carmen Maldonado-Bernal

**Affiliations:** 1 Programa de Doctorado en Ciencias Biomédicas, Universidad Nacional Autónoma de México, Mexico City, Mexico; 2 Laboratorio de Investigación en Inmunología y Proteómica, Hospital Infantil de México Federico Gómez, Mexico City, Mexico; 3 Departamento de Hematología, Hospital Pediátrico Moctezuma, Mexico City, Mexico; 4 Departamento de Hemato-Oncología, Hospital Infantil de México Federico Gómez, Mexico City, Mexico

**Keywords:** acute lymphoblastic leukemia, chemoresistance, cytotoxicity, immunomodulation, methotrexate, natural killer cells, P-glycoprotein (ABCB1), Toll-like receptor 2

## Abstract

**Introduction:**

Acute Lymphoblastic Leukemia (ALL) remains the most prevalent childhood malignancy. While chemotherapy has improved survival rates, multidrug resistance (MDR) mediated by P-glycoprotein (P-gp/ABCB1) overexpression persists as a major cause of treatment failure and relapse. Natural Killer (NK) cells are pivotal for anti-leukemic surveillance but are often compromised during treatment due to their susceptibility to chemotherapy, a vulnerability intrinsically linked to their own expression of P-gp. Strategies to transiently enhance NK cell chemoresistance could therefore preserve immune function and improve therapeutic outcomes. We hypothesized that stimulating Toll-Like Receptor 2 (TLR2) on NK cells could modulate P-gp expression or activity, enhancing their resilience to cytotoxic drugs.

**Methods:**

In this study, we first characterized NK cells from pediatric ALL patients, confirming the constitutive expression of both the therapeutic target (TLR2) and the drug efflux pump (P-gp) across all major subpopulations. Using a healthy donor model, we then dissected the functional consequences of specific TLR2 heterodimer engagement.

**Results:**

While agonists for both TLR2/1 (PCSK) and TLR2/6 (LTA, MALP-2) induced functional activation, their effects on P-gp were divergent. In a PBMC context, stimulation with the TLR2/1 agonist PCSK significantly enhanced the efflux of the chemotherapeutic agent Methotrexate (MTX), but not Rhodamine 123. This functional enhancement occurred without increasing P-gp surface expression, suggesting a modulation of transporter kinetics. Crucially, mechanistic assays in purified NK cells revealed that this MTX efflux enhancement relies on the cellular microenvironment, whereas direct high-dose TLR2/1 stimulation paradoxically led to P-gp loss. Furthermore, we demonstrated that the immunomodulatory effects of PCSK extend beyond chemoresistance to directly potentiate anti-leukemic effector functions; PCSK stimulation significantly enhanced NK cell cytotoxicity against RS4;11 leukemic blasts without compromising effector viability.

**Discussion:**

These findings identify a novel immunomodulatory axis where TLR2 signaling differentially regulates P-gp function and effector capacity in NK cells depending on the specific heterodimer engaged and the cellular context. We propose that controlled TLR2/1 stimulation represents a potential dual-benefit strategy to protect NK cells from chemotherapy-induced suppression while boosting their anti-leukemic activity in ALL.

## 1 Introduction

Acute Lymphoblastic Leukemia (ALL) remains the most prevalent childhood malignancy, representing a significant challenge in pediatric oncology (1, 2). While combination chemotherapy protocols have improved remission rates, the development of multidrug resistance (MDR) remains a primary obstacle to successful long-term treatment (3). A principal driver of this chemoresistance is the overexpression of the ATP-binding cassette (ABC) transporter, P-glycoprotein (P-gp, encoded by *ABCB1*), an efflux pump capable of expelling a wide range of cytotoxic drugs from the cell (4–6). Consequently, high P-gp expression on leukemic blasts is strongly correlated with patient relapse (7, 8), underscoring the urgent need for strategies to overcome this resistance mechanism (9).

Natural Killer (NK) cells are critical components of the innate immune system, uniquely equipped for the surveillance and elimination of malignant cells without prior antigen sensitization (10–13). However, the efficacy of NK cell-mediated anti-tumor immunity is often compromised during chemotherapy, as these effectors are highly susceptible to the systemic toxicity of cytotoxic agents. This vulnerability persists despite the fact that NK cells express the highest constitutive levels of P-gp among hematopoietic lineages (14–16). This creates a striking paradox: while leukemic blasts successfully leverage P-gp to achieve multidrug resistance (MDR) and evade treatment, the same defense mechanism appears insufficient to protect NK cells from collateral damage. Such a discrepancy suggests that, in the NK lineage, baseline protein presence does not necessarily equate to maximal functional efflux capacity (3). Interestingly, this has been identified as a unique 80 kDa “mini” variant rather than the classic 170 kDa form, though it retains similar substrate affinity (17). Consequently, while P-gp-mediated efflux allows cancer cells to survive in a toxic microenvironment, NK cells remain functionally impaired or depleted, undermining immune surveillance during therapy (18, 19).

Harnessing the intrinsic regulatory pathways of immune cells offers a novel therapeutic avenue to address this functional gap. NK cells express a functional repertoire of Pattern Recognition Receptors, including Toll-Like Receptors (TLRs) (20, 21). Specifically, TLR2 has been identified on the surface of human NK cells, where its engagement by ligands trigger critical effector functions, including IFNγ production and degranulation (22, 23). Crucially, a functional link between TLR2 signaling and the modulation of P-gp activity has been established in other lineages. Previous work demonstrated that TLR2 activation significantly increase P-gp-mediated efflux of methotrexate (MTX), thereby conferring resistance to the drug’s cytotoxic effects by enhancing transporter efficiency (24). This regulatory mechanism is potentially mediated through the p38-MAPK signaling pathway, a known component of TLR2 signaling that has also been implicated in the post-translational control of P-gp activity (25, 26).

While the TLR2-P-gp axis has been characterized in other lineages, its specific relevance and regulatory mechanism in human NK cells remain largely unexplored. Evidence from other cellular models suggests that P-gp activity can be modulated through two distinct pathways: the transcriptional induction of *de novo* protein expression or the post-translational enhancement of existing transporter kinetics, often involving phosphorylation or changes in subcellular localization (24, 25, 27). Given that NK cells already exhibit near-universal P-gp frequency, determining which of these regulatory mechanisms governs the TLR2-mediated response is critical for developing targeted chemoprotective strategies. Therefore, we hypothesized that direct TLR2 activation on NK cells would potentiate P-gp activity, leading to increased chemotherapeutic efflux and preserved anti-leukemic capacity. In this study, we investigated the direct and paracrine effects of TLR2 stimulation on P-glycoprotein function in human NK cells. We examined the differential effects of TLR2/1 and TLR2/6 agonists on functional activation and substrate-specific drug efflux, aiming to identify a strategy to protect immune effector cells during cancer therapy.

## 2 Individuals and methods

### 2.1 Patient recruitment and sample collection

Pediatric patients (aged 1–15 years) diagnosed with Acute Lymphoblastic Leukemia (ALL) were recruited from the Hospital Infantil de México Federico Gómez (HIMFG) and the Hospital Pediátrico Moctezuma. Inclusion criteria comprised a confirmed diagnosis of *de novo* ALL. To ensure no prior exposure to cytotoxic agents, peripheral blood samples were collected in EDTA-coated tubes at the time of hospital admission, prior to the initiation of any chemotherapy, corticosteroid therapy, or induction protocol. Furthermore, participants had no history of recent blood transfusion or active infection. The study protocol was approved by the Ethics and Research Committees of the HIMFG (Protocol No. HIM-2023-036), and written informed consent was obtained from the parents or legal guardians, along with assent from the children when applicable, in accordance with the Declaration of Helsinki.

### 2.2 Cell isolation and culture

Peripheral Blood Mononuclear Cells (PBMCs) were isolated from patient blood samples and from waste buffy coats of healthy adult donors (aged 18–60 years). Waste buffy coats were provided by the HIMFG Blood Bank through a formal institutional agreement regulated by the Research and Ethics Committees. The use of adult donors as a healthy comparative baseline is supported by evidence that the fundamental immunophenotype and constitutive P-gp expression of NK cells remain consistent between healthy pediatric and adult populations (15, 18).

PBMC were isolated by density gradient centrifugation using Lymphoprep™ (STEMCELL Technologies, Vancouver, Canada). Briefly, blood was diluted 1:2 (venous blood) or 1:4 (buffy coat) with sterile saline and centrifuged at 800 x g for 30 minutes at 20 °C. The PBMC layer was collected, washed twice with saline, and resuspended in RPMI-1640 medium (Corning, NY, USA; Cat: 10-040-CVR) supplemented with 10% Fetal Bovine Serum (FBS; Gibco, Waltham, MA, USA; Cat: 16000-044).

For experiments requiring purified NK cells, cells were isolated from PBMCs by negative selection using the EasySep™ Human NK Cell Isolation Kit (STEMCELL Technologies, Vancouver, Canada; Cat: 17955) according to the manufacturer’s instructions. Purity was routinely assessed by flow cytometry (CD3-CD56+), typically exceeding 90%. Cells were cultured in RPMI-1640 supplemented with 10% FBS at 37 °C in a 5% CO2 humidified atmosphere.

### 2.3 TLR2 ligand stimulation

To assess TLR2-mediated activation, cells (PBMCs or purified NK cells, 1x10^6 cells/mL) were stimulated for 24 hours with the following specific agonists:

- PCSK (Pam3Cys-SK4, TLR2/1 agonist): Enzo Life Sciences, Farmingdale, NY, USA (Cat: 165-066-m002).
- LTA (Lipoteichoic Acid from *Streptococcus faecalis*, TLR2/6 agonist): AbD Serotec/Bio-Rad, Kidlington, UK (Cat: 0100-0035).
- MALP-2 (Macrophage-activating lipopeptide-2, TLR2/6 agonist): Enzo Life Sciences, Farmingdale, NY, USA (Cat: ALX-162-027).

Lyophilized agonists were reconstituted, stored, and utilized strictly according to the manufacturers’ safety and product information guidelines. PCSK and MALP-2 are synthetic lipopeptides certified as high-purity ligands, inherently free of other TLR agonists or endotoxin contamination. All compounds were reconstituted in sterile endotoxin-free water or PBS. Untreated cells (UT) received an equivalent volume of the sterile vehicle and were maintained under identical conditions (including incubation time and Brefeldin A treatment) to serve as the baseline negative control. Stimulation was performed using dose-response curves (0.01 – 20 µg/mL for PCSK/LTA; 1–100 ng/mL for MALP-2) to determine optimal activation conditions (24).

### 2.4 Flow cytometry and immunophenotyping

Cell surface staining was performed using fluorochrome-conjugated monoclonal antibodies incubated for 30 minutes at 4 °C in the dark. To ensure optimal staining quality and adhere to standardized flow cytometry best practices, all incubation and wash steps were performed using a staining buffer supplemented with 1% Bovine Serum Albumin (BSA) (Sigma Aldrich A7906) to minimize non-specific binding and maintain cell viability. The following antibodies were obtained from BioLegend (San Diego, CA, USA): anti-CD3 Pacific Blue (Clone UCHT1), anti-CD19 PE-Cy7 (Clone HIB19), anti-CD16 PerCP-Cy5.5 (Clone 3G8), anti-CD56 PE (Clone MEM-188) or APC (Clone HCD56), anti-TLR2 PE/APC (Clone TL2.1), anti-ABCB1/P-gp PE (Clone UIC2), and anti-NKG2D APC-Cy7 (Clone 1D11).

NK cells were identified through a standardized sequential gating strategy. Initial events were gated by light-scattering properties (FSC vs. SSC) to identify the lymphocyte population, followed by FSC-A versus FSC-H gating to exclude doublets and cell aggregates. Within the singlet lymphocyte gate, T and B cells were excluded (CD3+/CD19+). Total NK cells were then defined as CD3–CD19– lymphocytes expressing the classic markers CD56 and CD16. To enhance the stringency of the identification and capture the cytotoxic effector lineage, NKG2D expression was included as a complementary marker (28, 29).

For targeted analysis of P-gp modulation, the identified NK cell population was further stratified into three distinct functional subsets based on the density of CD56 and CD16: CD56+CD16– (Regulatory-associated), CD56+CD16+ (Cytotoxic), and CD56–CD16+ (ADCC-associated). Total NK cell data represent the integrated sum of these three subpopulations. Notably, CD56–CD16– double-negative cells were excluded from all subsequent functional and phenotypic assessments to ensure high population specificity. For experiments utilizing enriched NK cells, purity was consistently >95%.

For intracellular cytokine (IFNγ) detection, cells were treated with Brefeldin A (BioLegend; Cat: 420601) for the last 6 hours of the 24-hour stimulation period to arrest protein transport and allow for the accumulation of even constitutive cytokine pools. Cells were then fixed, permeabilized using the Cytofix/Cytoperm™ kit (BD Biosciences, San Jose, CA, USA), and stained with anti-IFNγ PE-Cy7 (Clone 4S.B3; BioLegend). Unstained cells and Fluorescence Minus One (FMO) controls were utilized to establish accurate detection thresholds and define the positive staining population. Degranulation was assessed by the surface expression of CD107a; anti-CD107a FITC antibody (Clone H4A3; BioLegend) was added to the culture medium during the stimulation period. Data were acquired using a CytoFLEX LX flow cytometer (Beckman Coulter, Brea, CA, USA) or a Cytek Aurora spectral cytometer (Cytek Biosciences, Fremont, CA, USA). Data analysis was performed using FlowJo software v10.10 (Tree Star/BD Biosciences).

### 2.5 P-glycoprotein functional efflux assays

P-gp activity was evaluated using fluorescent substrate efflux assays. Cells (1x10^6) were loaded with either Rhodamine 123 (Rh123, 150 ng/mL; Sigma-Aldrich, St. Louis, MO, USA) or Methotrexate-FITC (MTX-FITC) in RPMI medium for 30 minutes at 37 °C. While MTX-FITC stock solutions were initially prepared in DMSO, they were further diluted to ensure negligible final concentrations (<0.01% v/v) in the culture. Preliminary experiments confirmed that the vehicle, including these concentrations of DMSO, did not induce activation or alter P-gp efflux in NK cells. Following the loading phase, cells were washed twice with ice-cold PBS to arrest transport.

For the efflux phase, cells were resuspended in warm efflux medium (RPMI + 10% FBS) in the presence or absence of the specific P-gp inhibitor Verapamil (100 µM; Sigma-Aldrich, Cat: PHR1131-16) and incubated for 60 minutes at 37 °C. Cells were then washed with ice-cold PBS and analyzed by flow cytometry.

### 2.6 Dual assessment of NK cell cytotoxicity: ldh release and flow cytometry

To robustly evaluate the effect of TLR2 stimulation on NK cell cytotoxic function, we employed a dual-readout assay. Purified NK cells (Effectors, E) from healthy donors (n=3) were co-cultured with RS4;11 leukemic blasts (Targets, T; ATCC, Manassas, VA, USA) in 48-well plates. Cells were seeded at an Effector: Target (E:T) ratio of 0.5:1 (50,000 NK cells: 100,000 RS4;11 cells) in a final volume of 500 µL of phenol red-free RPMI-1640 supplemented with 2% FBS. Co-cultures were treated with PCSK (10 µg/mL), LTA (10 µg/mL), or left untreated (UT) and incubated for 24 hours at 37 °C in 5% CO2.

For the LDH release assay (CytoTox 96^®^ Non-Radioactive Cytotoxicity Assay; Promega, Madison, WI, USA; Cat: G1780), appropriate controls were included for each donor. Following incubation, samples were centrifuged at 400 x *g* for 5 minutes to prevent mechanical lysis. 50 µL of the cell-free supernatant was transferred to a 96-well enzymatic assay plate and incubated with CytoTox 96^®^ Reagent for 30 minutes in the dark. Absorbance was measured at 490 nm using a microplate reader. The percentage of specific cytotoxicity was calculated as per manufacturer instructions.

For the flow cytometric assessment, the cell pellets remaining from the LDH harvest were stained with Viakrome 808 Fixable Viability Dye (Beckman Coulter) to identify dead cells, followed by surface staining with anti-CD19-PE-Cy7 (Targets), anti-CD56-APC, and anti-CD16-PE (Effectors). The RS4;11 target population was specifically gated as CD19+, while effector NK cells were identified as the CD19– fraction. Cytotoxicity was quantified by gating on the CD19+ population and calculating the percentage of Viakrome 808+ cells.

### 2.7 Data analysis and statistics

The efflux capacity was quantified using the Pumping Index (PI), calculated utilizing the Median Fluorescence Intensity (MFI) according to the formula:


$$PI=\frac{MFI_{Inhibitor}}{MFI_{Efflux}}$$

A PI > 0 indicates active drug efflux. The MFI was prioritized for activity calculation due to its higher sensitivity for detecting graded kinetic changes, although the percentage of fluorescent-positive cells was also analyzed as a complementary metric (30–32).

Statistical analysis was performed using GraphPad Prism version 10.6.1 (GraphPad Software, Boston, MA, USA). Data distribution was assessed for normality. Comparisons between treatment groups were performed using Two-way Repeated Measures (RM) ANOVA followed by Dunnett’s multiple comparisons test to compare treatment conditions against the untreated control. A *p*-value< 0.05 was considered statistically significant.

## 3 Results

### 3.1 Immunophenotypic characterization of NK cell subpopulations in pediatric ALL patients

A total of 11 pediatric patients diagnosed with Acute Lymphoblastic Leukemia (ALL) were enrolled in this study. The baseline clinical and demographic characteristics for this cohort are summarized in **Table 1**. The median age of the patients was 5 years (range: 1–15 years), and the majority (10/11) presented with a Pre-B immunophenotype.

**Table 1:** Baseline characteristics of pediatric ALL patients.

General characteristics of patients	
N° of cases	11
Sex M:F	7:4
Age media (range)	5 (1-15) years
Immunophenotype	
Pre B	10
B	1

Summary of demographic and clinical data for the study cohort (n=11). Data for age at diagnosis are presented as the median value followed by the range (minimum–maximum) in parentheses. All other data are presented as patient counts. *ALL, Acute Lymphoblastic Leukemia; n, number of patients; Pre-B, pre-B cell precursor.*

**Figure 1:**
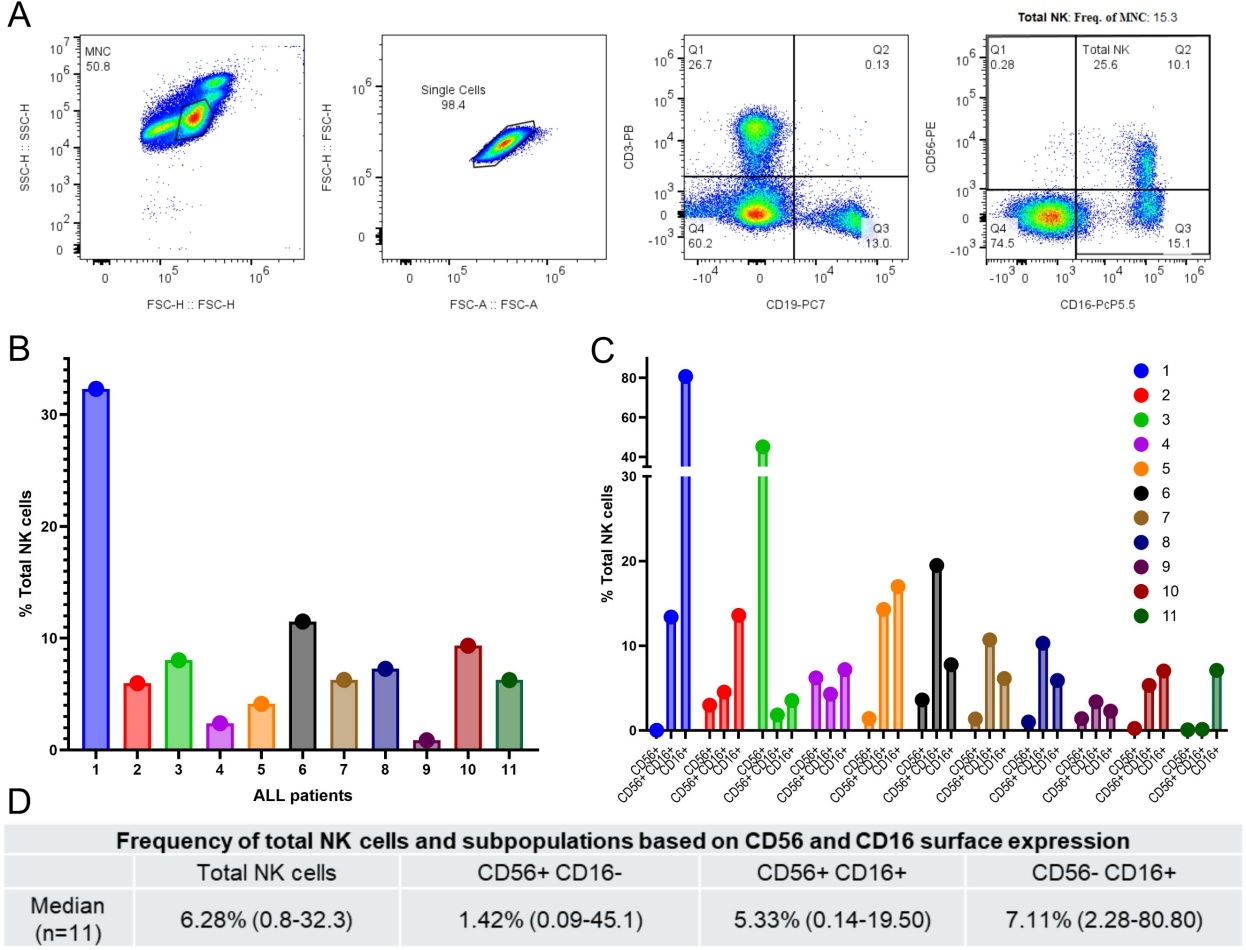
Flow cytometric identification and characterization of NK cell subpopulations in pediatric ALL patients. **(A)** Representative gating strategy used to identify NK cells and their subsets in PBMCs. Cells were first identified by light-scattering properties (FSC-H vs. SSC-H) to gate the mononuclear cell (MNC) population, followed by doublet exclusion (FSC-A vs. FSC-H) to identify singlets. Lineage-negative cells (CD3-PacificBlue/CD19-PE/Cy7) where then selected to exclude T and B lymphocytes. NK cells were subsequently identified and subclassified based on the expression of CD56-PE and CD16-PerCP/Cy5.5 into three distinct subsets: Q1 (CD56+CD16-, Regulatory), Q2 (CD56+CD16+, Cytotoxic), and Q3 (CD56-CD16+, ADCC-associated). Total NK cells were defined as the sum of these three subpopulations (Q1 + Q2 + Q3), excluding the CD56-CD16- double-negative population (Q4). **(B)** Frequency of total NK cells relative to total PBMCs for each of the 11 pediatric ALL patients enrolled. **(C)** Distribution of the three NK cell subpopulations (as defined in A) for each individual patient. **(D)** Summary table showing the median frequency and range (minimum–maximum) for the Total NK cell population and each subpopulation across the cohort (n=11).

To characterize the NK cell compartment in these patients, we employed a multiparametric flow cytometry gating strategy (**Figure 1A**). After excluding doublets and lineage-positive cells (CD3+/CD19+), NK cells were identified and further stratified into three functional subpopulations based on their CD56+ and CD16- expression profiles: CD56+CD16-, CD56+CD16+, and CD56-CD16+. Total NK cells were defined as the integrated sum of these three subsets, systematically excluding the CD56–CD16– double-negative (DN) population. This DN fraction represents a heterogeneous, unassigned population likely consisting of immature precursors or residual non-NK cells that lack definitive mature effector markers, a phenomenon particularly relevant in the leukemic microenvironment (19, 33).

Analysis of the total NK cell frequency revealed significant heterogeneity within the patient cohort, with a median frequency of 6.28% of total PBMCs (**Figure 1B**). We further dissected the distribution of the specific subpopulations (**Figure 1C**). The CD56-CD16+ subset represented the most abundant subpopulation in this cohort (median 7.11%), followed by the double-positive CD56+CD16+ subset (median 5.33%) and the CD56+CD16- subset (median 1.42%) (**Figure 1D**). This characterization confirms the presence of all major functional NK cell subsets in the peripheral blood of pediatric ALL patients.

### 3.2 Pediatric ALL patient NK cells constitutively express the therapeutic target and the drug efflux pump (ABCB1)

**Figure 2:**
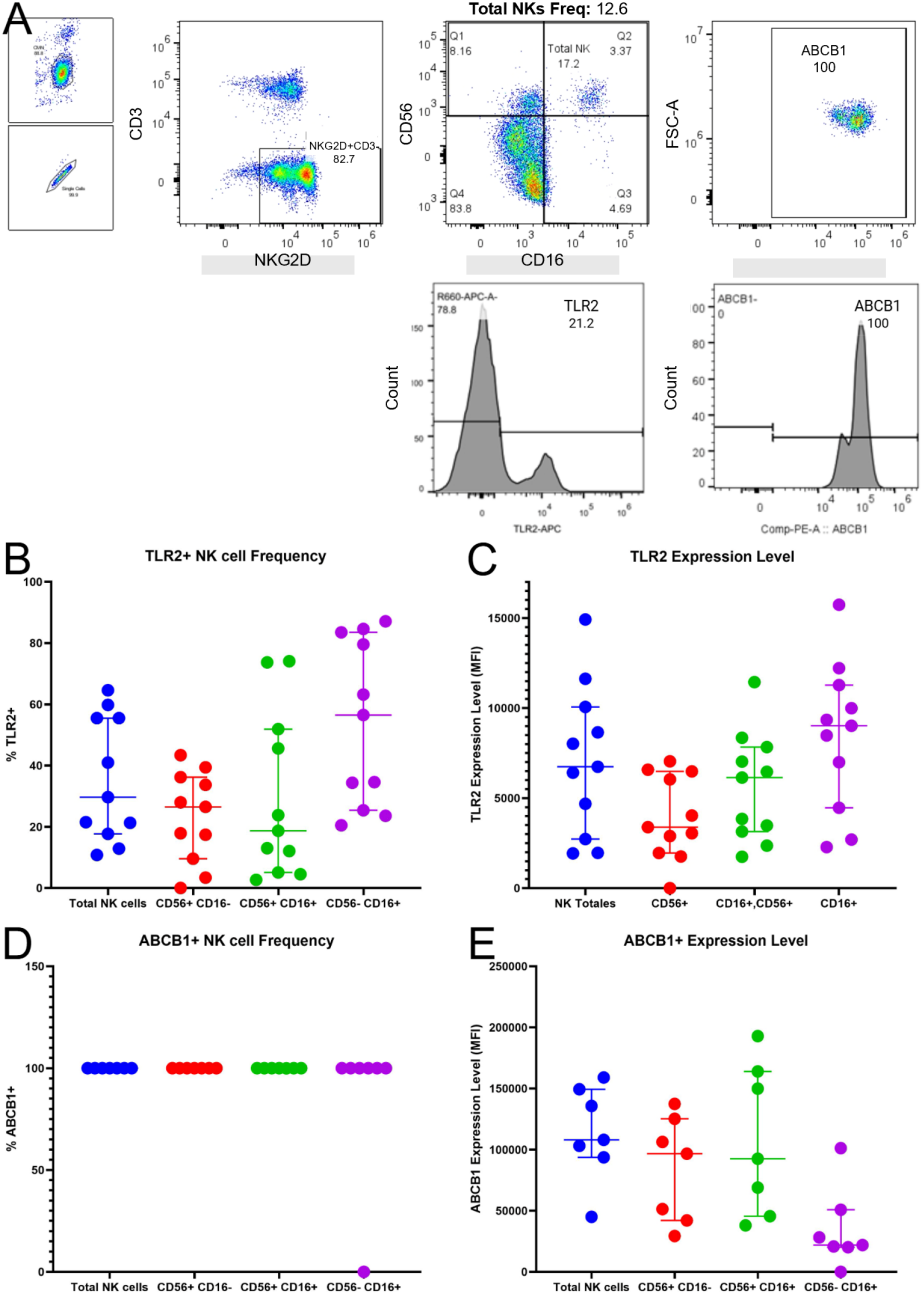
Baseline expression of P-glycoprotein (ABCB1) and TLR2 on NK cells from pediatric ALL patients. Surface expression of the drug efflux pump (P-gp) and the therapeutic target (TLR2) was assessed in patient samples. **(A)** Representative flow cytometry gating strategy. Total NK cells were identified through sequential gating: To ensure population specificity for functional analysis, NK cells were identified as CD3–NKG2D+ events. NK subpopulations were further defined based on CD16 and CD56 expression. Histograms show representative staining for TLR2 and ABCB1 (P-gp) on the Total NK cell population. **(B, C)** Expression of TLR2 analyzed in the full patient cohort (n=11), shown as **(B)** Frequency (% positive) and **(C)** Magnitude (MFI). **(D, E)** Expression of ABCB1 (P-glycoprotein) analyzed in a subgroup of patients (n=7), shown as **(D)** Frequency (% positive) and **(E)** Magnitude (MFI). Each dot represents an individual patient; horizontal lines indicate the median and interquartile range.

Having defined the NK cell subpopulations, we next validated the presence of the specific molecular components required for our proposed immunomodulation strategy. We assessed the expression of the receptor TLR2 and the multidrug resistance protein P-glycoprotein (P-gp/ABCB1) in patient samples using a refined gating strategy (**Figure 2A**). Unlike the diagnostic lineage-exclusion approach (CD3–/CD19–) used for the general characterization in **Figure 1**, this analysis utilized NKG2D as a confirmatory positive marker to ensure that subsequent protein quantification was restricted to mature NK effector cells.

We first assessed the expression of TLR2, the target for our immunomodulatory ligands, in the full patient cohort (n=11). TLR2 was detected on NK cells from all patients, though with notable heterogeneity. The median frequency of TLR2+ cells within the Total NK population was approximately 30%, with expression present across all three subpopulations (**Figure 2B**). Similarly, the magnitude of expression (MFI) varied among patients but confirmed the presence of the receptor at the protein level (**Figure 2C**).

Next, we evaluated the baseline expression of P-glycoprotein (ABCB1). In a representative subgroup of patients (n=7), we found that P-gp expression was ubiquitous. Strikingly, the frequency of ABCB1+ cells was near 100% for Total NK cells and the CD56+ and CD56+CD16+ subsets in virtually all patients analyzed (**Figure 2D**). While the protein was universally present, the density of expression (MFI) varied between individuals (**Figure 2E**). These data demonstrate that circulating NK cells in pediatric ALL patients constitutively express the necessary molecular machinery for the TLR2-mediated P-gp modulation hypothesized in this study.

### 3.3 Characterization of the healthy donor experimental model

While the patient data confirms the clinical presence of TLR2 and P-gp, establishing the mechanistic link between TLR2 signaling and P-gp modulation requires a robust and reproducible experimental system. Therefore, all subsequent functional and mechanistic studies were conducted using NK cells isolated from healthy adult donors.

**Figure 3:**
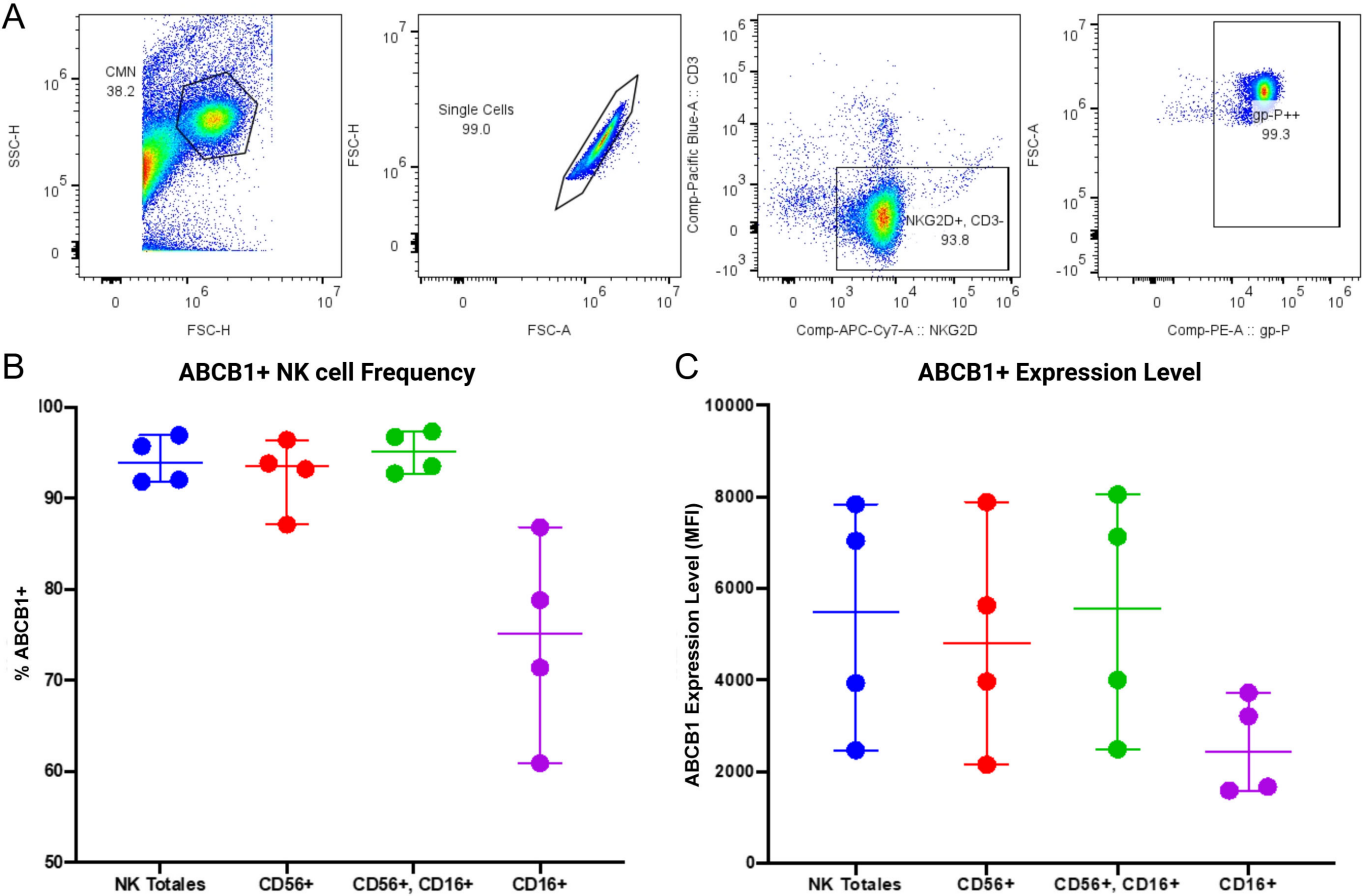
Baseline P-glycoprotein (P-gp) surface expression on NK cell subpopulations from healthy donors. To establish the experimental model, baseline P-gp expression was measured on resting NK cells isolated from healthy adult donors (n=4). **(A)** Representative flow cytometry histograms showing P-glycoprotein (ABCB1) expression on Total NK cells and the three defined subpopulations. **(B)** Frequency of P-gp+ cells within Total NK cells and the subpopulations. **(C)** Magnitude of P-gp expression, shown as Median Fluorescence Intensity (MFI). The high basal expression (>90%) in most subsets validates the model for efflux modulation studies. Each dot represents an individual donor; horizontal lines indicate the median and interquartile range.

We first validated the baseline expression of P-glycoprotein in this healthy model (**Figure 3**). Representative flow cytometric analysis confirms that P-gp is constitutively expressed on the surface of resting NK cells (**Figure 3A**). Consistent with our observations in the patient cohort, the frequency of P-gp+ cells was notably high (>90%) in the Total NK, CD56+, and CD56+CD16+ populations (**Figure 3B**). The CD16+ subset displayed a slightly lower and more variable frequency of expression compared to the other subsets. In terms of protein density, the MFI of P-gp showed expected inter-donor variability (**Figure 3C**). This confirms that our healthy donor model constitutively expresses P-gp, validating its suitability for evaluating modulation strategies.

### 3.4 TLR2 ligation induces robust functional activation of NK Cells in a PBMC context

**Figure 4:**
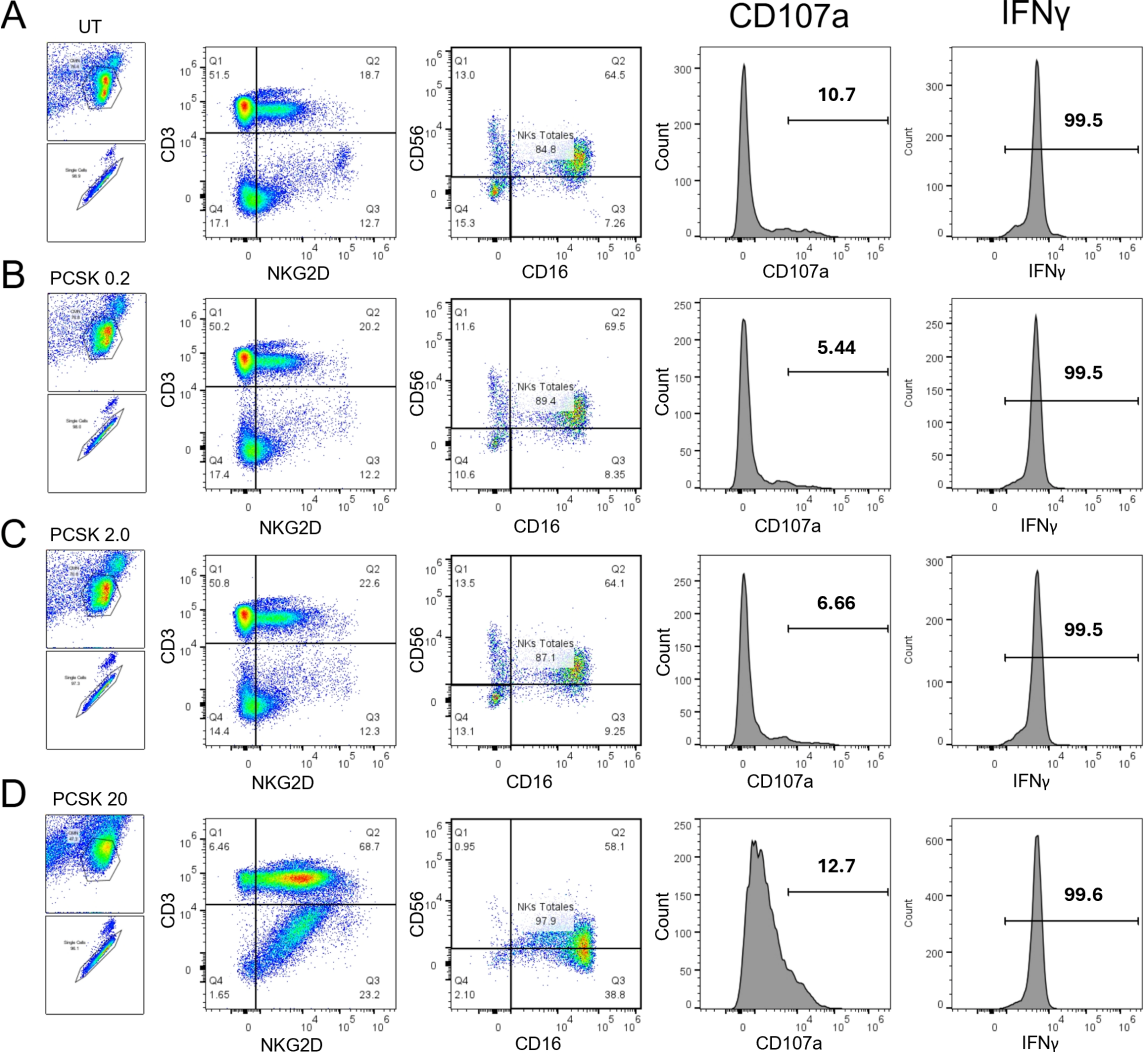
Representative flow cytometric analysis of NK cell functional activation following TLR2 stimulation. PBMCs from healthy donors were cultured for 24 hours with increasing concentrations of the TLR2 ligand PCSK or left untreated (UT). Rows represent the specific stimulation conditions: **(A)** Untreated control, **(B)** 0.2 µg/mL, **(C)** 2.0 µg/mL, and **(D)** 20.0 µg/mL. Columns display the sequential gating strategy: identification of total NK cells (CD3-NKG2D+) and subsequent assessment of functional markers. The final density plots show CD107a (y-axis) vs. IFNγ (x-axis). Note the dose-dependent emergence of CD107a+ and IFNγ+ populations (upper quadrants) in row D compared to the control in **(A)** This gating and analysis strategy was applied similarly for LTA and MALP-2 stimulation experiments.

We next verified the functional competency of the TLR2 signaling pathway in our model. PBMCs were stimulated with increasing concentrations of three distinct TLR2 ligands: PCSK (TLR2/1), LTA (TLR2/6), and MALP-2 (TLR2/6). Representative flow cytometric analysis for PCSK (**Figure 4**) shows a visible, dose-dependent activation, with distinct populations of CD107a+ and IFNγ+ NK cells emerging as the concentration increased from 0.2 to 20.0 µg/mL.

**Figure 5:**
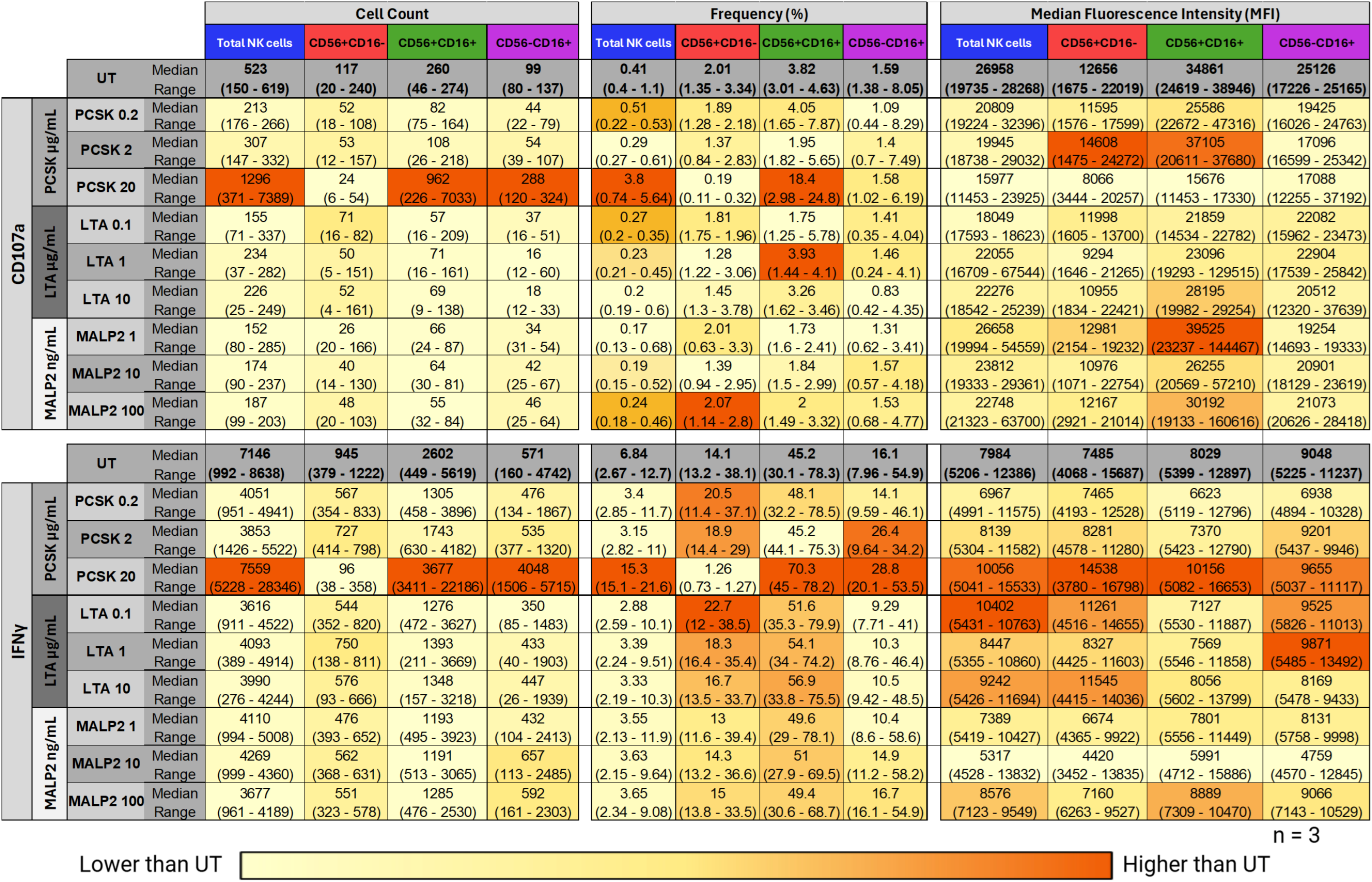
Heatmap of NK cell functional responses in PBMCs following TLR2 ligand stimulation. PBMCs from healthy donors (n=3) were stimulated for 24 hours with increasing concentrations of PCSK (TLR2/1 agonist), LTA (TLR2/6 agonist), and MALP-2 (TLR2/6 agonist), or left untreated (UT). Functional responses were assessed by flow cytometry for CD107a (top panel) and IFNγ (bottom panel). Data are presented as the Median followed by the Range (minimum–maximum) for three parameters: Cell Count, Frequency (%), and Median Fluorescence Intensity (MFI). Columns correspond to Total NK cells and the three defined subpopulations (CD56+CD16–, CD56+CD16+, and CD56–CD16+). The color scale reflects the median value relative to the UT control, ranging from light yellow (lower than UT) to dark orange (higher than UT). UT, Untreated control.

The functional response for all ligands, summarized in a heatmap (**Figure 5**), revealed clear, ligand-specific activation patterns. PCSK induced the broadest response, showing a consistent increase across all metrics for both CD107a and IFNγ, particularly in the Total NK and CD56+CD16+ subsets. Interestingly, the TLR2/6 ligands LTA and MALP-2 displayed more selective profiles. While they induced only variable changes in CD107a, both ligands triggered a robust, dose-dependent increase in IFNγ production. This suggests differential signaling outcomes between TLR2/1 and TLR2/6 pathways in NK cells within the PBMC environment.

### 3.5 TLR2 stimulation drives functional NK cell activation through both direct and paracrine pathways

**Figure 6:**
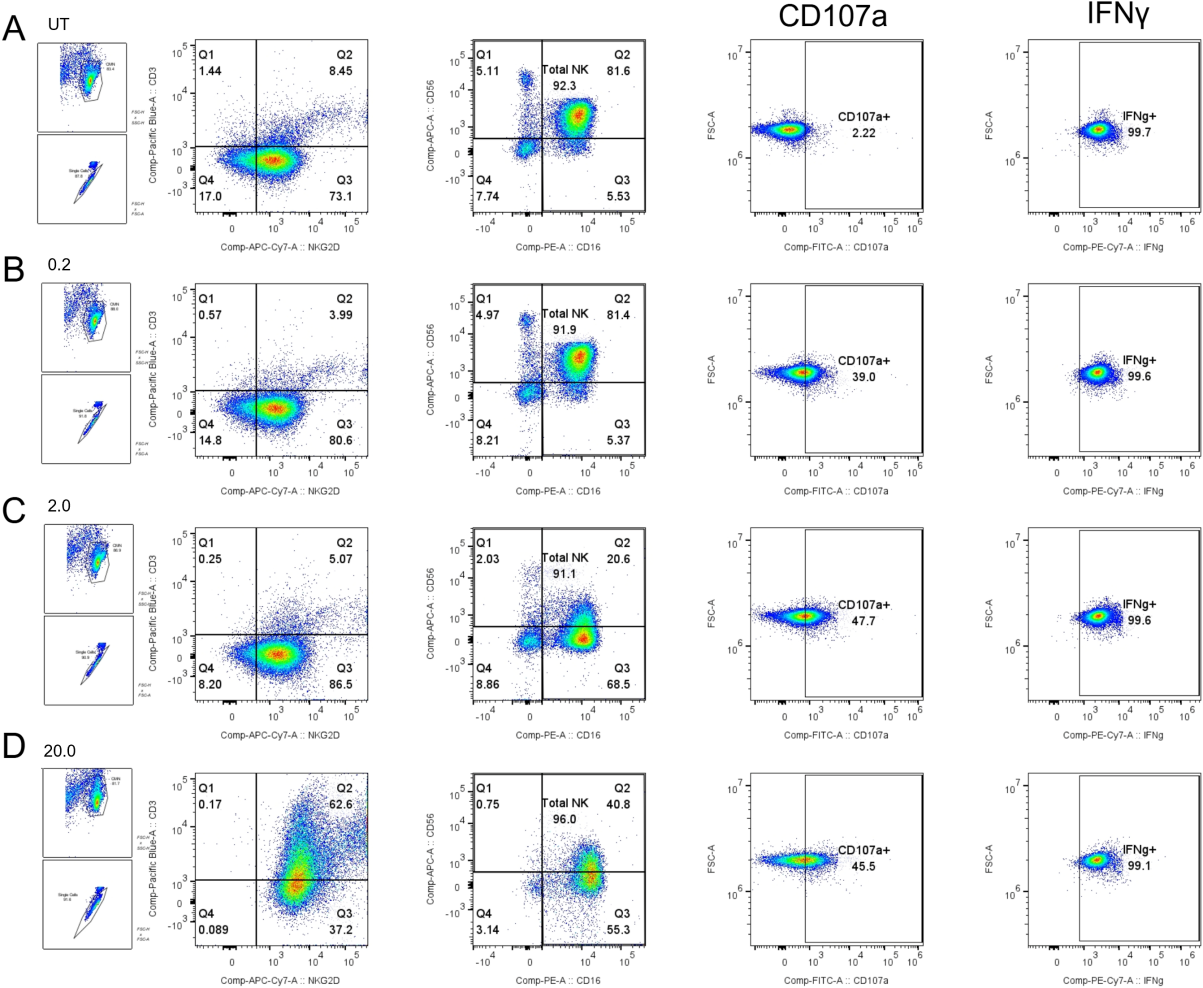
Representative flow cytometric analysis of functional activation in purified NK cells following TLR2 stimulation. Purified NK cells isolated from healthy donors were cultured for 24 hours with increasing concentrations of the TLR2 ligand PCSK (0.2, 2.0, and 20.0 µg/mL) or left untreated (UT). Rows represent the specific stimulation conditions: **(A)** Untreated control (UT), **(B)** 0.2 µg/mL, **(C)** 2.0 µg/mL, and **(D)** 20.0 µg/mL. Columns display the gating strategy: identification of mononuclear cells and single cells, confirmation of the CD3– NKG2D+ NK cell phenotype, identification of subsets via CD56 and CD16 expression, and subsequent assessment of functional markers (CD107a and IFNγ). Note the direct induction of CD107a+ and IFNγ+ populations even in the absence of neighboring cells.

**Figure 7:**
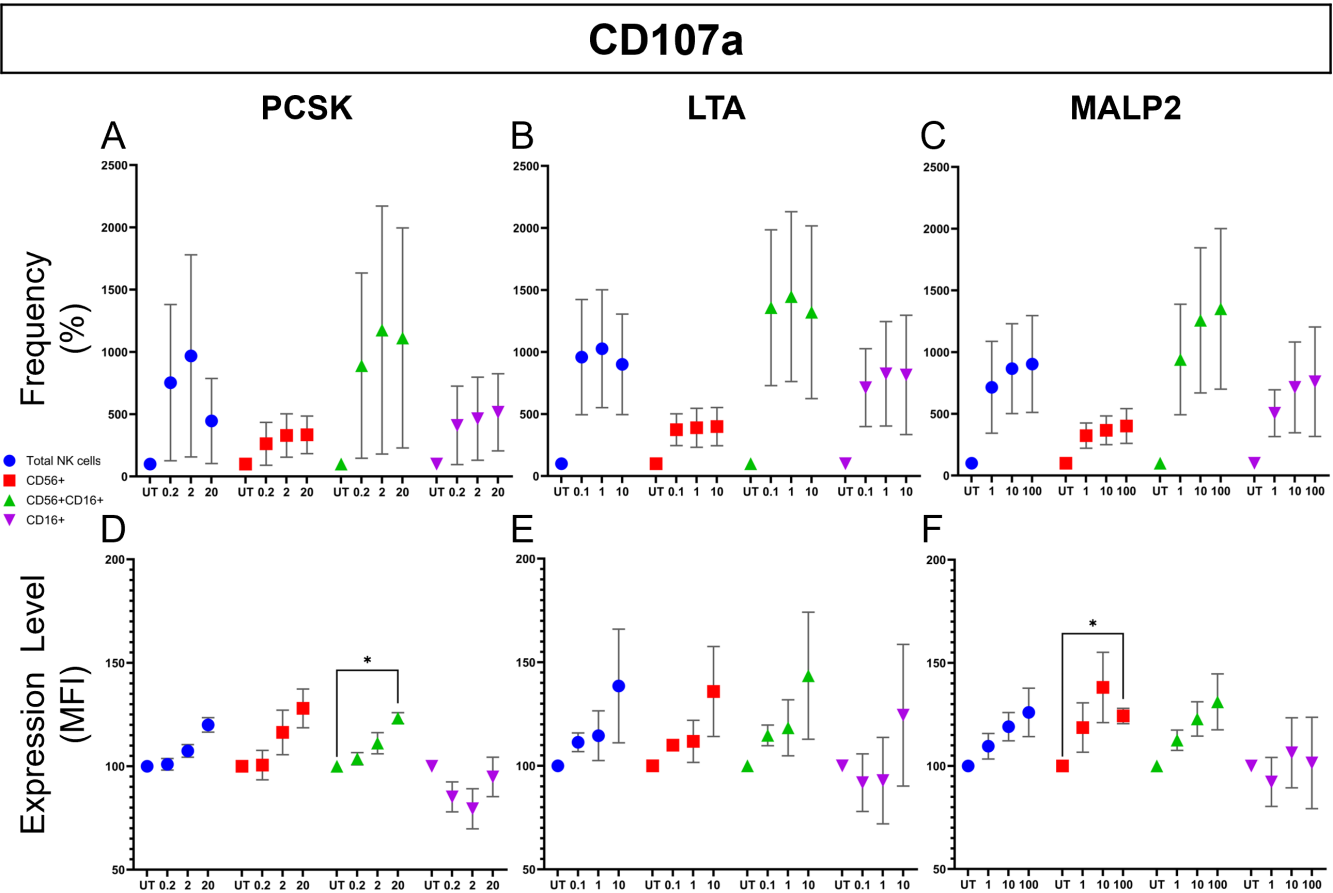
Direct effect of TLR2 ligands on NK cell degranulation (CD107a expression). Purified NK cells isolated from healthy donors (n=3) were stimulated for 24 hours with increasing concentrations of PCSK (Left column), LTA (Middle column), or MALP-2 (Right column). Degranulation was assessed by flow cytometry measuring surface CD107a (LAMP-1) expression. Top row **(A–C)** shows the Frequency (% positive cells) and bottom row **(D–F)** shows the Magnitude (MFI) of expression. Note on gating: “Total NKs” (blue circles) represents the frequency relative to the parental CD3-NKG2D+ population. The subpopulations (CD56+ [red squares], CD56+CD16+ [green triangles], and CD16+ [purple inverted triangles]) represent the frequency within the Total NK cell gate. All data were normalized to the untreated control (UT, 100%) and are presented as mean ± SEM. Statistical analysis was performed using a two-way repeated measures (RM) ANOVA followed by Dunnett’s multiple comparisons test. Asterisks indicate statistical significance compared to the UT control (p< 0.05). Significant increases in CD107a MFI were observed with PCSK in the CD56+CD16+ subset **(D)** and with MALP-2 in the CD56+ subset **(F)**. * means p<0.05.

**Figure 8:**
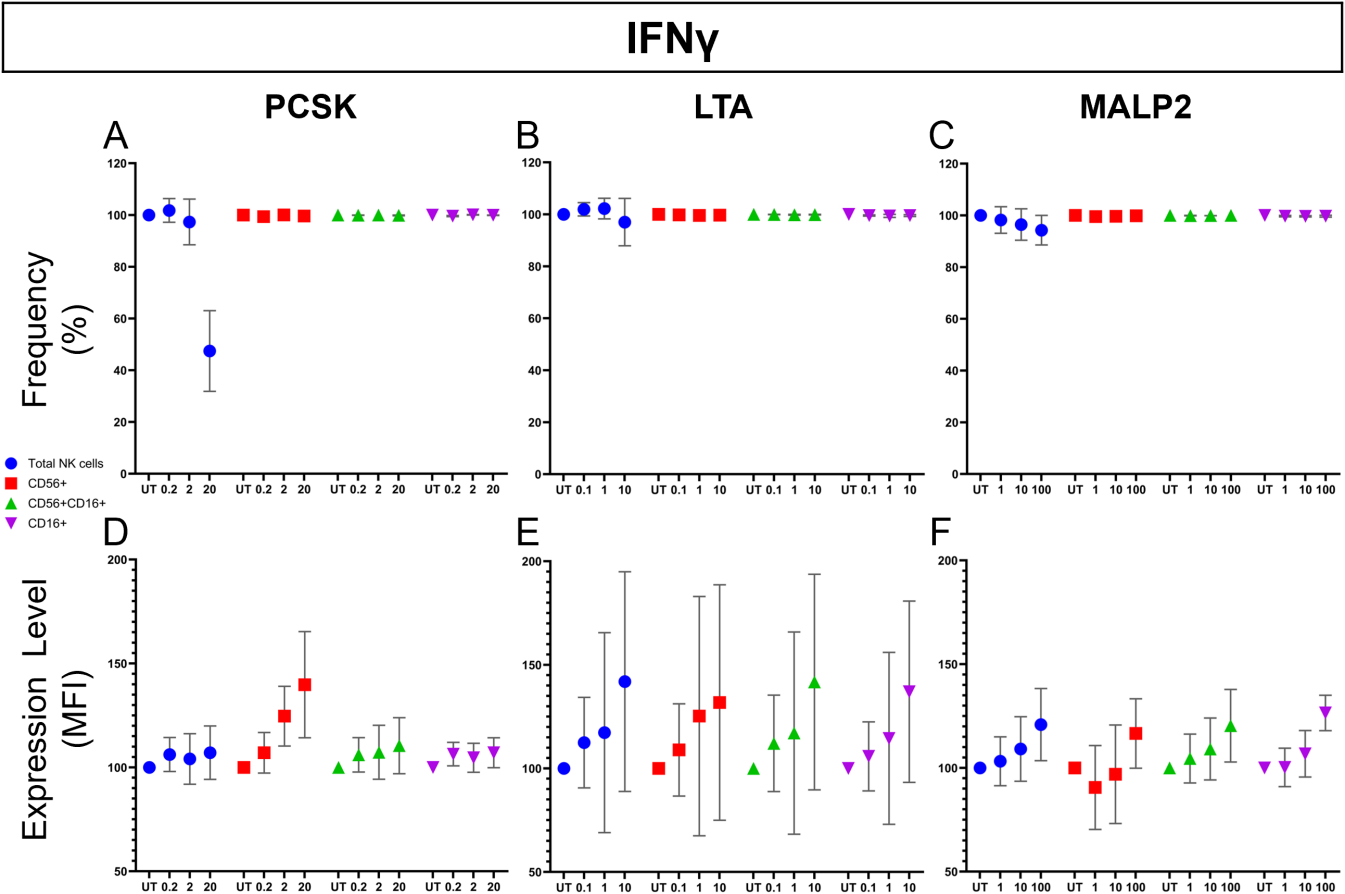
Direct effect of TLR2 ligands on NK cell cytokine production (IFNγ expression). Purified NK cells isolated from healthy donors (n=3) were stimulated for 24 hours with increasing concentrations of PCSK (left column), LTA (middle column), or MALP-2 (right column). Brefeldin A was added during the final 6 hours of incubation to arrest protein transport and facilitate intracellular cytokine accumulation. IFNγ expression was assessed by multiparametric flow cytometry. **(A–C)** Frequency of IFN**γ**+ cells presented on a unified scale (0–120%). Subpopulations (CD56+CD16–, red; CD56+CD16+, green; and CD56–CD16+, purple) and total NK cells (blue) are expressed relative to parental population. **(D–F)** Magnitude of expression quantified by Median Fluorescence Intensity (MFI) on a unified scale (50–200). Note that IFN**γ** frequency is constitutively high (>95%) across all functional subsets in the untreated control (UT). All data were normalized to the UT (100%) and are presented as mean ± SEM. Statistical analysis was performed using a two-way repeated measures (RM) ANOVA. No statistically significant differences were observed (p > 0.05), although a dose-dependent increasing trend in MFI was noted, particularly in the regulatory-associated subset (CD56+CD16–) following PCSK stimulation **(D)**.

To distinguish between direct NK cell activation and neighboring cell-mediated (paracrine) effects, we repeated the stimulation assays using highly-purified NK cells. Representative flow cytometric analysis for PCSK (**Figures 6A–D**) provides visual confirmation that TLR2 ligation can directly trigger effector functions in the absence of other cell populations. We then quantified these responses across all ligands and donors to dissect the specific pathways involved (**Figures 7**, **8**).

We first analyzed the degranulation (CD107a) response evaluating both the frequency of positive cells and the magnitude of expression across all subsets (**Figures 7A–F**). Regarding the frequency of CD107a+ NK cells, stimulation with the three TLR2 ligands generally induced a dose-dependent upward trend compared to the untreated control (**Figures 7A–C**). We then analyzed the magnitude of degranulation by Median Fluorescence Intensity (MFI) (**Figures 7D–F**). Stimulation with PCSK (TLR2/1) induced a clear, dose-dependent upward trend in CD107a MFI across all subsets, reaching statistical significance in the CD56+CD16+ subset at the highest concentration (20 µg/mL) (**Figure 7D**). In contrast, LTA (TLR2/6) stimulation did not induce any observable significant change in CD107a expression (**Figure 7E**). Finally, MALP-2 (TLR2/6) elicited a dose-dependent increase in degranulation magnitude, which was statistically significant in the CD56+ subset (**Figure 7F**).

Next, we examined IFNγ production in isolated NK cells (**Figure 8**). Under unstimulated conditions (UT), the baseline frequency of IFNγ+ cells was constitutively high (>95%) across all three functional subsets (CD56+CD16–, CD56+CD16+, and CD56–CD16+; red, green, and purple dots, respectively) (**Figures 8A–C**). In contrast, the frequency of total NK cells (blue dots), calculated relative to the total parental population, was notably lower and exhibited a dose-dependent decline at higher ligand concentrations. This downward trend was particularly abrupt following stimulation with 20 µg/mL of PCSK (**Figure 8A**) and appeared progressive with increasing doses of MALP-2 (**Figure 8C**).

The magnitude of the cytokine response was further quantified by Median Fluorescence Intensity (MFI) (**Figures 8D–F**). Despite the high baseline frequency, a visible, dose-dependent upward trend in IFNγ MFI was observed across all three ligands and subpopulations compared to the UT control. Notably, the CD56+CD16– subset exhibited the most consistent elevation in MFI following PCSK stimulation (**Figure 8D**). While these increases did not reach statistical significance (p > 0.05), a consistent stepwise elevation in the median MFI values confirms that direct TLR2 ligation is sufficient to independently trigger a basal level of cytokine production. However, the contrast between the robust response in PBMCs and this attenuated magnitude in isolated cells highlights that, while not absolute for initiation, integrated intercellular crosstalk—involving both soluble factors (paracrine) and contact-dependent (juxtacrine) signals—is required to reach the optimal magnitude of activation observed in systemic environments (34, 35).

### 3.6 Analysis of P-glycoprotein activity and surface expression in human NK cells across different substrates and cellular microenvironments

#### 3.6.1 Modulation of P-glycoprotein activity for rhodamine 123

Having confirmed functional activation, we addressed the central hypothesis of this study: modulation of P-glycoprotein activity. We first evaluated P-gp function using Rhodamine 123 (Rh123), a classic fluorescent substrate.

**Figure 9:**
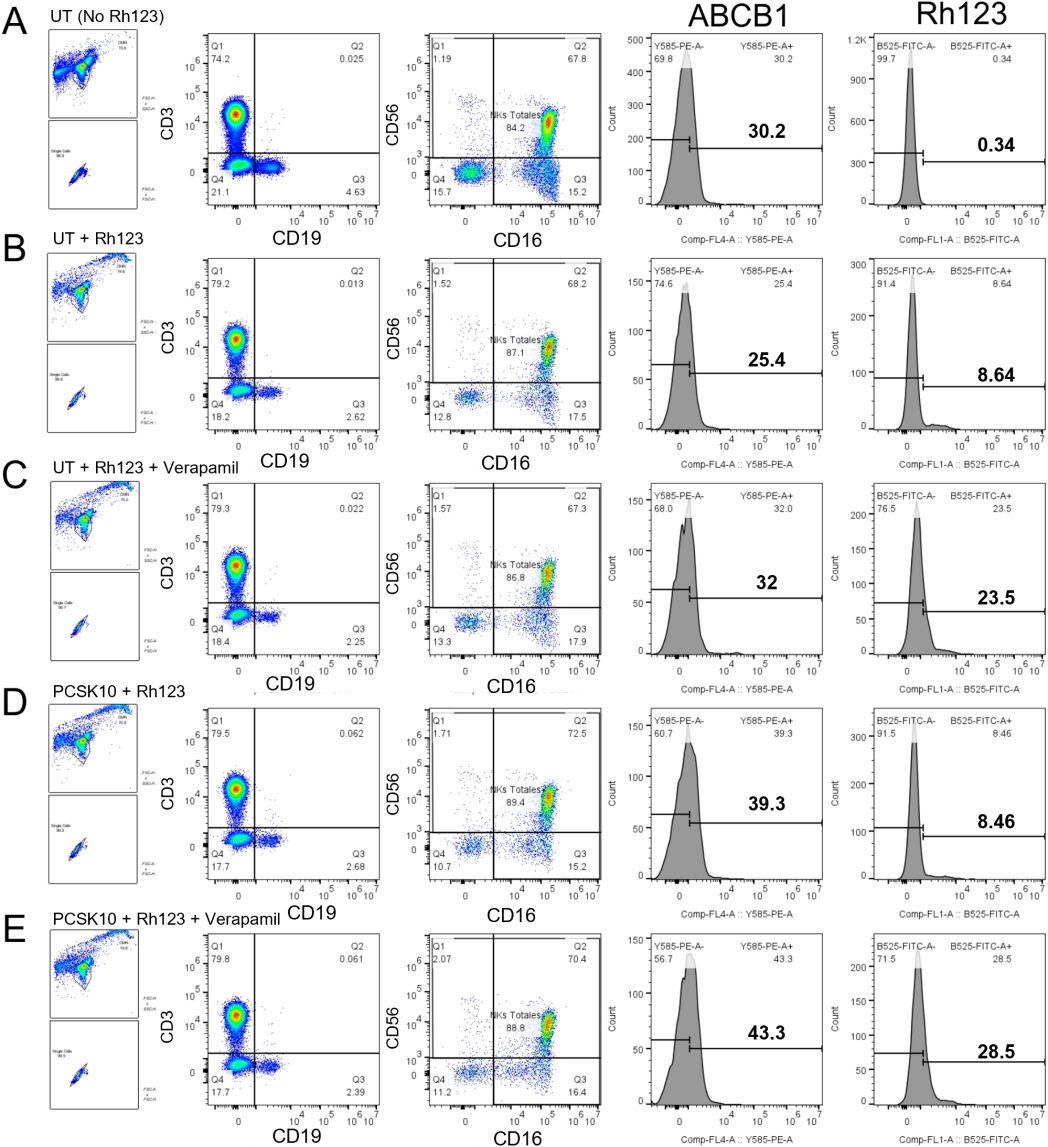
Representative flow cytometric analysis of Rhodamine 123 efflux and P-glycoprotein inhibition in NK cells. PBMCs were subjected to a functional efflux assay to validate P-gp activity. Rows represent specific assay conditions: **(A)** Unstained cells (Autofluorescence control). **(B)** Untreated cells loaded with Rhodamine 123 (Rh123) followed by a 60-minute efflux period (Baseline efflux). **(C)** Untreated cells loaded with Rh123 + efflux in the presence of the P-gp inhibitor Verapamil (100 µM) (Maximum retention control). **(D)** Cells stimulated with PCSK (10 µg/mL) + Rh123 efflux. **(E)** Cells stimulated with PCSK + Rh123 efflux + Verapamil. The histograms display the fluorescence intensity of Rh123 (FITC channel) in the gated NK cell population. Note the shift to higher fluorescence (increased retention) in the Verapamil-treated conditions, confirming active P-gp-mediated transport.

To validate the assay sensitivity and specificity, we analyzed the efflux profiles by flow cytometry (**Figure 9**). Crucially, the addition of the specific P-gp inhibitor Verapamil blocked dye efflux, resulting in a marked retention of Rh123 and a shift to higher fluorescence intensity (**Figure 9C**), confirming that the observed efflux is indeed mediated by P-gp.

**Figure 10:**
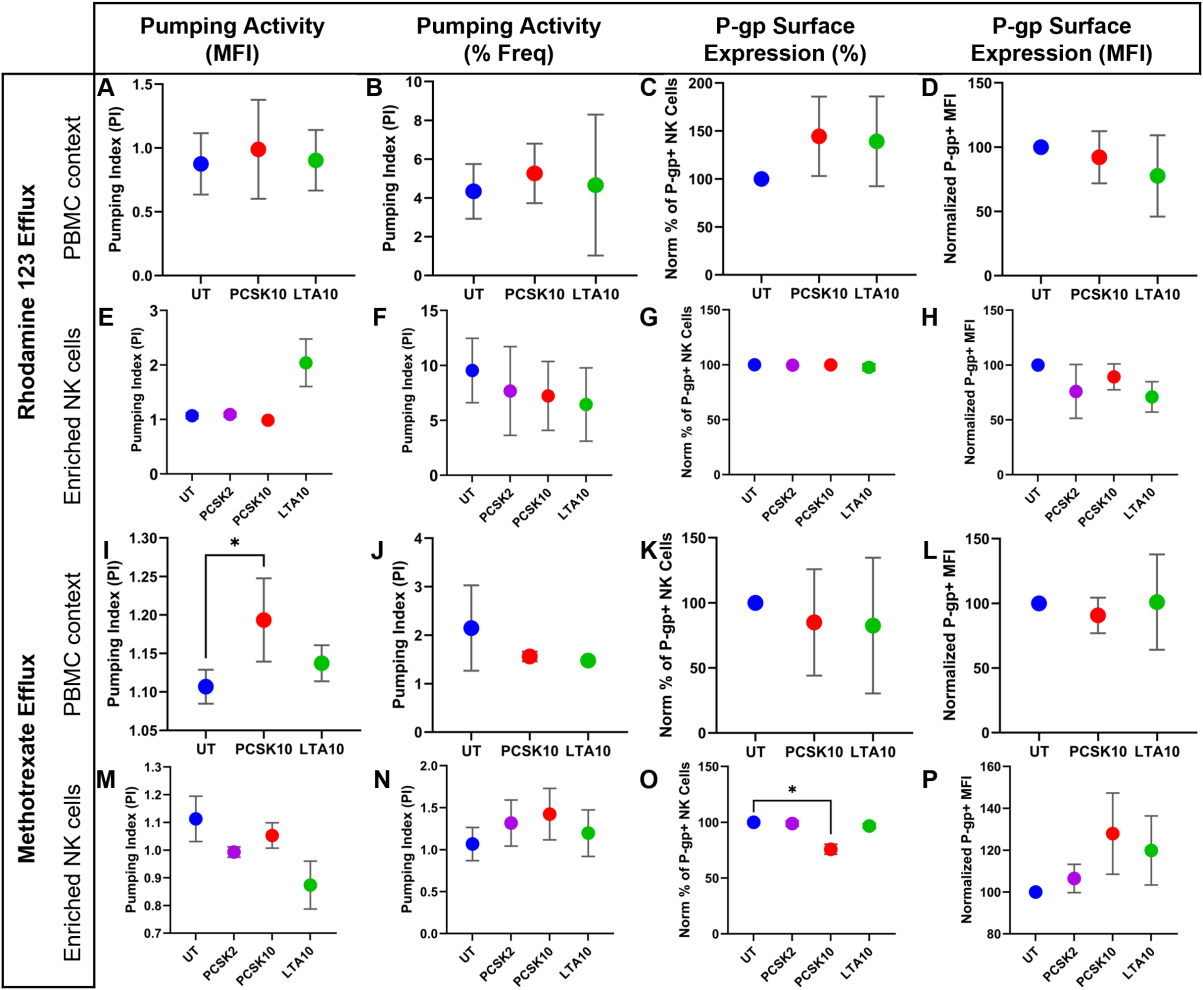
Comparative analysis of P-glycoprotein (P-gp) activity and surface expression in human NK cells across different substrates and cellular microenvironments. Comprehensive assessment of P-gp (ABCB1) function and expression in NK cells identified by flow cytometry as CD3-NKG2D+ and further characterized by the expression of CD56 and CD16. Cells were stimulated for 24 hours within total PBMCs (n=3) or as enriched populations (n=3) with the TLR2/1 agonist PCSK (10 µg/mL), the TLR2/6 agonist LTA (10 µg/mL), or left untreated (UT). The 16-panel matrix is organized by substrate and context: Rhodamine 123 (Rh123) efflux in PBMCs **(A–D)** and enriched NK cells **(E–H)**, and Methotrexate-FITC (MTX-FITC) efflux in PBMCs **(I–L)** and enriched NK cells **(M–P)**. For each condition, the panels display the Pumping Index (PI) derived from the Median Fluorescence Intensity (MFI) **(A, E, I, M)**, the frequency of substrate-positive cells **(B, F, J, N)** (a PI > 1.0 indicates active efflux), the normalized frequency of P-gp+ cells **(C, G, K, O)**, and the normalized P-gp MFI **(D, H, L, P)**. In enriched NK cell assays **(E–H, M-P)**, a low-dose PCSK condition (2 μg/mL, violet data points) was included to evaluate dose-dependent regulatory trends. Statistical significance was observed for the enhancement of MTX-FITC efflux in the PBMC context following PCSK stimulation (Panel I) and the reduction in P-gp surface frequency under direct high-dose stimulation (Panel O). Data are presented as mean ± SEM. Statistical analysis was performed using a repeated measures one-way ANOVA followed by Dunnett’s multiple comparisons test (p< 0.05 vs. UT).

We then quantified these responses in the total PBMC context (**Figure 10**, Row 1). Stimulation with either PCSK (10 µg/mL) or LTA (10 µg/mL) did not induce a statistically significant change in P-gp pumping activity for Rh123, regardless of whether the Pumping Index (PI) was calculated by MFI (**Figure 10A**) or frequency (**Figure 10B**). Regarding P-gp protein levels, while stimulation appeared to induce an upward trend in the frequency of P-gp+ cells (**Figure 10C**) and a downward shift in MFI (**Figure 10D**), these changes did not reach statistical significance. This lack of significance is likely attributed to the high inter-donor variability and the limited sample size (n=3) utilized for these functional assays.

However, when assayed in enriched NK cells (**Figure 10**, Row2), a distinct functional trend emerged. For these experiments, we included a lower dose of PCSK (2 µg/mL, violet points) to detect subtle dose-dependent effects. While PCSK remained ineffective at both doses, stimulation with LTA (10 µg/mL) induced a marked increase in the Pumping Index calculated by MFI (mean PI increased to ~2.0) (**Figure 10E**), but not in frequency (**Figure 10F**). Although not statistically significant due to variability, the magnitude suggests a potential direct enhancement of transport activity by TLR2/6 signaling that is masked in the PBMC environment. Intriguingly, this trend was accompanied by a noticeable reduction in P-gp surface expression (**Figures 10G, H**).

#### 3.6.2 Substrate-specific potentiation of methotrexate efflux in PBMCs

To determine clinical relevance, we assessed the modulation of efflux for the chemotherapeutic agent Methotrexate (MTX).We returned to the PBMC context to assess if the broad activation profile of PCSK observed earlier translated into functional drug resistance (**Figure 10**, Row 3). In striking contrast to the Rh123 results, stimulation with the TLR2/1 agonist PCSK (10 µg/mL) induced a statistically significant increase in the Pumping Index (PI) calculated by MFI compared to the untreated control (**Figure 10I**). This indicates that activation of the TLR2/1 signaling axis significantly enhances the overall capacity of NK cells to expel this specific chemotherapeutic agent within a multicellular enviroment. While the PI calculated by frequency showed a similar trend, it did not reach significance (**Figure 10J**). Importantly, this functional enhancement occurred without changes in P-gp protein expression (**Figures 10K, L**).

#### 3.6.3 Direct TLR2 stimulation induces P-gp loss rather than enhanced activity in isolated NK cells

To determine if the PCSK-driven enhancement of Methotrexate (MTX) efflux observed in the PBMC context was a direct effect, we performed the assay on enriched NK cells (**Figure 10**, Row 4). The results in the isolated system stood in sharp contrast to the PBMC data. Direct stimulation with either PCSK or LTA failed to increase the Pumping Index (PI) for Methotrexate-FITC (**Figures 10M, N**). Notably, stimulation with LTA (10 µg/mL) induced a marked and significant reduction in the magnitude of the PI (**Figure 10M**), suggesting a functional impairment of the efflux machinery under direct TLR2/6 engagement in isolation.

Mechanistic analysis revealed a divergent regulatory pattern between protein frequency and density. We observed a statistically significant decrease in the frequency of P-gp+ NK cells following stimulation with PCSK at 10 µg/mL (**Figure 10O**). Interestingly, the P-gp MFI showed an opposite, albeit non-significant, upward trend in the remaining positive population (**Figure 10P**). This discrepancy suggests that the loss of surface P-gp may preferentially affect NK cells with lower basal expression levels, thereby shifting the median fluorescence of the surviving P-gp+ population toward higher values.

These findings indicate that while the paracrine environment in PBMCs facilitates increased P-gp activity, direct and potent TLR2 stimulation in isolation leads to a reduction in surface P-gp frequency. This phenomenon of transporter loss, potentially through internalization or proteasomal degradation as documented in other models under intense cellular stress (36–38), underscores the critical role of cell-to-cell crosstalk in sustaining P-gp-mediated chemoresistance (34, 35).

### 3.7 PCSK stimulation potentiates NK cell cytotoxicity against leukemic blasts without compromising effector viability

Finally, to determine if the functional activation and P-gp modulation described above translate into enhanced anti-leukemic potential, we performed *in vitro* cytotoxicity assays against the B-cell precursor ALL cell line RS4;11.

**Figure 11:**
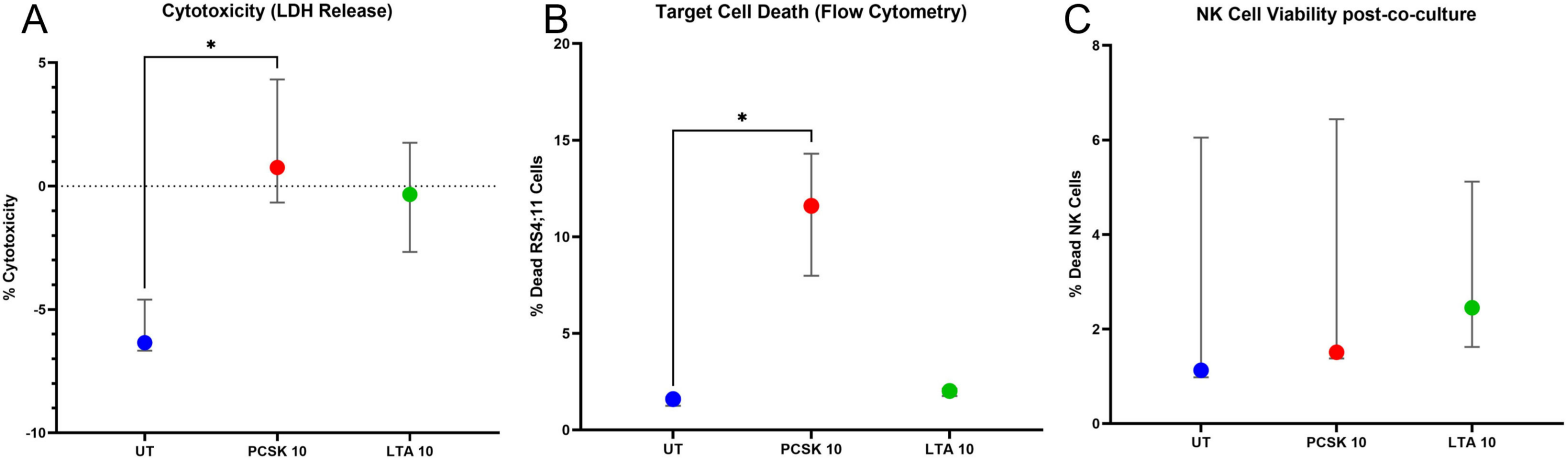
TLR2/1 stimulation selectively enhances NK cell anti-leukemic cytotoxicity without compromising effector cell viability. Comprehensive evaluation of the anti-leukemic potential and safety profile following TLR2 stimulation. Purified NK cells from healthy donors (n=3) were co-cultured with RS4;11 leukemic blasts at an effector:target (E:T) ratio of 0.5:1 for 24 hours under stimulation with PCSK (10 µg/mL), LTA (10 µg/mL), or left untreated (UT). Anti-leukemic activity was quantified through a dual-readout strategy: **(A)** LDH release assay, representing specific lysis calculated from enzymatic activity in the supernatant, and **(B)** flow cytometric analysis of target cell death, shown as the frequency of CD19+ Viakrome808+ events. **(C)** Effector cell survival was simultaneously monitored within the same co-culture system by measuring the frequency of dead total NK cells (defined as CD56 and/or CD16 positive populations; Viakrome808+). Data are presented as mean ± SEM. Statistical analysis was performed using a repeated measures one-way ANOVA followed by Dunnett’s multiple comparisons test (*p< 0.05 vs. UT).

We employed a dual-readout strategy to robustly quantify target cell death. First, measuring LDH release in the supernatant revealed that stimulation with PCSK (10 µg/mL) significantly increased the specific lysis of RS4;11 leukemic cells compared to the untreated control (**Figure 11A**). In contrast, LTA stimulation did not induce a significant increase in cytotoxicity over baseline.

To corroborate this finding and confirm target specificity, we analyzed the cells by flow cytometry. Gating specifically on the leukemic targets (CD19+), we quantified cell death using a viability dye. We observed a significant increase in the frequency of dead cells (Viakrome 808+) in the PCSK-treated co-cultures (**Figure 11B**), mirroring the enzymatic LDH results.

Importantly, to ensure that the treatment was not cytotoxic to the effector cells themselves, we assessed the viability of the total NK cells (CD56+ and/or CD16+) within the same co-culture wells (**Figure 11C**). The frequency of dead NK cells remained consistently low (<3%) across all conditions, with no significant difference between treated and untreated groups. This confirms that the increased killing observed with PCSK is due to enhanced effector function against the leukemia, rather than non-specific toxicity or NK cell death.

## 4 Discussion

### 4.1 Differential signaling of TLR2 heterodimers dictates NK cell functional outcomes

The central finding of this study is that the functional activation of human NK cells and the subsequent modulation of P-glycoprotein (P-gp) activity are strictly dependent on the specific TLR2 heterodimer engaged. While the expression of P-gp in NK cells has been previously documented, its functional regulation following immune activation remains largely unexplored (39, 40). Our study addresses this gap, revealing that the TLR2/1 agonist PCSK induces a broad, direct activation profile, whereas the TLR2/6 agonists (LTA and MALP-2) elicit responses heavily reliant on the cellular microenvironment. This functional divergence aligns with the established biochemistry of TLR2, which recruits distinct adaptor molecules and signaling kinetics depending on whether it heterodimerizes with TLR1 or TLR6 (41–43).

### 4.2 The intracellular vs. direct dichotomy in cytokine production

Our data revealed that TLR2/6 stimulation (LTA and MALP-2) triggered a potent IFNγ response in the PBMC context that was markedly attenuated in isolated NK cells. This suggests that the robust proinflammatory response typically associated with TLR2/6 signaling involves a complex intercellular crosstalk with neighboring cell populations. This interaction likely encompasses both the release of accessory cytokines, such as IL-12 or IL-18 from monocytes (44, 45), and direct cell-to-cell contact, which has been shown to provide critical co-stimulatory signals for optimal TLR-mediated NK cell activation (34, 35). In contrast, the TLR2/1 agonist PCSK maintained a direct, dose-dependent induction of IFNγ in isolated cells. While the neighboring microenvironment is required to reach maximal activation magnitudes, our results confirm that direct TLR2 ligation is sufficient to independently trigger a functional response, highlighting the presence of a functional, autonomous TLR2 signaling pathway in human NK cells (22). Notably, the high constitutive frequency (~100%) of IFNγ+ cells observed in unstimulated (UT) isolated NK cells likely reflects the intracellular accumulation of basal cytokine pools. Brefeldin A treatment arrests protein transport, allowing even minimal constitutive production to reach detectable levels, particularly when utilizing high-sensitivity antibodies. This baseline saturation confirms that the isolated population consists of functionally capable effectors primed for response.

### 4.3 Mechanistic implications for P-gp modulation: signaling and post-translational regulation

A pivotal discovery of this work is the ability of TLR2/1 signaling to enhance the efflux of the chemotherapeutic agent Methotrexate (MTX). This finding extends the work of Frank et al., who originally described a TLR2-p38 MAPK axis capable of modulating P-gp-mediated MTX resistance in myeloid cells and intestinal epithelium (24). Our results demonstrate that this mechanism is conserved in human NK cells but is strictly regulated by the ligand specificity and the cellular context. Specifically, we observed a divergence between P-gp protein levels and functional activity in the PBMC context; while TLR2 stimulation led to a trend toward increased frequency of P-gp+ cells alongside a subtle decrease in MFI, MTX efflux was significantly enhanced.

This apparent discordance suggests that P-gp activity is modulated through post-translational regulation rather than simple protein over-expression. The human P-gp transporter contains a central “linker” region (residues 656–689) that serves as a specific substrate for Protein Kinase C (PKC) (46). Since TLR2 heterodimers are known to selectively activate PKC isoforms (47), this pathway likely triggers the phosphorylation of key serine residues (Ser-661, Ser-667, and Ser-671) within the linker domain. Such phosphorylation acts as a fine-tuning mechanism that optimizes the pump’s ATPase activity and substrate transport kinetics. Consequently, the increased pumping efficiency observed—even when surface MFI remains stable or shows a downward trend—reflects an optimized kinetic state of the existing pumps.

This mechanism is particularly relevant when comparing the basal landscape of pediatric ALL patients versus healthy donors. While healthy NK cells exhibit a frequency of ~94%, NK cells from ALL patients reach a near-universal basal expression frequency (~100%). This constitutive state could suggests that the leukemic microenvironment may further upregulate P-gp expression, leading to the near-saturation of the transporter across all subsets even before the initiation of therapy.

Conversely, LTA (TLR2/6) stimulation in isolated cells “unmasked” a trend toward increased pumping efficiency for Rhodamine 123. This aligns with the association of TLR2/6 with pro-survival and cell expansion signals in hematopoietic progenitors (48). It is plausible that TLR2/6 engagement triggers post-translational modifications, such as phosphorylation, that enhance the kinetic rate of the P-gp pump to support cell survival, a mechanism that appears to be suppressed by paracrine signals in the full PBMC environment. Furthermore, the specific transport of MTX but not Rh123 by PCSK-stimulated cells highlights the complexity of P-gp substrate recognition. However, the balance is delicate. While PCSK enhanced MTX efflux in PBMCs, it led to a significant loss of surface P-gp upon direct high-dose stimulation in isolated NK cells. In the absence of the protective paracrine microenvironment, potent TLR2/1 activation likely seems to trigger the internalization or degradation of the transporter through over-phosphorylation or proteasomal turnover, a phenomenon previously documented in other models under intense oxidative or pharmacological stress (36–38).

### 4.4 Methodological considerations in assessing P-gp activity

To functionally characterize the TLR2-mediated modulation of P-glycoprotein, we utilized flow cytometric efflux assays with both Rhodamine 123 (Rh123) and Methotrexate (MTX). In quantifying efflux, we prioritized the Median Fluorescence Intensity (MFI) to calculate the Pumping Index. This approach aligns with consensus guidelines suggesting that MFI provides superior sensitivity and reproducibility for detecting graded changes in transporter kinetics compared to the percentage of positive cells, which relies on arbitrary thresholding (30–32).

This prioritization is critical given the functional dynamics observed in our results; as TLR2 stimulation enhances the magnitude of substrate efflux across the existing P-gp pool, the resulting shift in fluorescence intensity is accurately captured by the PI. In contrast, the frequency of positive cells remains insensitive to such functional modulation, especially in clinical contexts where NK cells have already exhibit a ubiquitous basal expression frequency. Indeed, our results with PCSK in PBMCs showed a statistically significant enhancement in pumping activity when measured by MFI, reflecting a generalized increase in pump efficiency across the NK cell population rather than the recruitment of a specific subset. However, we retained the analysis of frequency as a complementary metric, which proved critical in revealing the subtle recruitment of P-gp+ cells within the PBMC environment and the dramatic loss of surface expression in isolated cells under high-dose stimulation.

This dual-metric approach was equally vital in interpreting our cytokine data. While frequency was technically saturated at baseline (near 100% for IFNγ and CD107a), MFI allowed us to resolve the specific, dose-dependent increases in cytokine magnitude induced by TLR2 ligands. Moreover, tracking the frequency of total NK cells relative to the singlet population revealed a dose-dependent decline at high ligand concentrations. This suggests that potent, direct TLR2/1 activation may reach a threshold that triggers activation-induced cell death or the proteolytic shedding of lineage markers—phenomena that MFI alone would fail to capture, highlighting the delicate balance required to harness TLR2 for therapeutic benefit. Enhanced Cytotoxicity and Clinical Relevance

Crucially, we demonstrated that the immunomodulatory effects of PCSK extend beyond chemoresistance to directly potentiate anti-leukemic effector functions. PCSK stimulation significantly enhanced NK cell cytotoxicity against RS4;11 leukemic blasts, as confirmed by both LDH release and direct target cell viability assays. Importantly, this enhanced killing occurred without compromising the viability of the effector NK cells themselves. This dual benefit—potentially protecting NK cells from chemotherapy via P-gp potentiation while simultaneously boosting their cytotoxic activity against blasts—positions TLR2/1 agonists as promising candidates for adjuvant immunotherapy in ALL. The constitutive expression of P-gp on NK cells from pediatric ALL patients described here underscores the clinical barrier to effective immunotherapy during chemotherapy. However, the balance is delicate; as our data show, excessive direct stimulation may lead to transporter loss. Future therapies must carefully calibrate TLR2 engagement to harness the protective efflux capacity without compromising cell viability or inducing exhaustion.

## 5 Conclusion

In this study, we provide the first comprehensive characterization of the functional link between specific TLR2 heterodimers and P-glycoprotein activity in human NK cells. We demonstrate that NK cells from pediatric ALL patients constitutively express the necessary molecular machinery (TLR2 and P-gp) to be targeted by this immunomodulatory strategy. Our data reveal a complex, heterodimer-specific regulation: while TLR2/6 agonists primarily drive paracrine-dependent cytokine production, the TLR2/1 agonist PCSK triggers a robust direct cytotoxic response and selectively enhances the efflux of the chemotherapeutic agent Methotrexate.

Crucially, we dissected the mechanism of this modulation, showing that the enhancement of drug efflux relies on the cellular microenvironment, whereas direct high-dose stimulation can lead to transporter loss. Most importantly, this modulation does not come at the cost of effector function; rather, TLR2/1 stimulation significantly potentiates NK cell cytotoxicity against leukemic blasts while maintaining effector viability. These findings propose a dual therapeutic benefit for TLR2/1 agonist in ALL: protecting NK cells from chemotherapy-induced suppression via P-gp potentiation and simultaneously boosting their anti-leukemic activity. Future translational studies should focus on optimizing dosing regimens to balance these direct and paracrine effects in the clinical setting.

## Data Availability

The raw data supporting the conclusions of this article will be made available by the authors, without undue reservation.

## Supplementary material

Supplementary Figure 1 The illustration depicts (Left) the high constitutive frequency of P-glycoprotein (P-gp) expression observed in NK cells from pediatric patients with acute lymphoblastic leukemia (ALL). (Right) it shows how TLR2 engagement triggers an intracellular signaling cascade (proposed mechanism) that optimizes P-gp efflux kinetics and boosts anti-leukemic effector functions, such as IFN-gamma secretion and CD107a degranulation, leading to enhanced cytotoxicity against leukemic blasts. This visual summary clarifies that TLR2 signaling acts as a functional booster to bridge the gap between basal protein expression and effective chemoresistance.
