# Supplementary figures and images for: TLR2 activation potentiates P-glycoprotein-mediated methotrexate efflux and enhances cytotoxicity of human NK cells against acute lymphoblastic leukemia
Source: Front Immunol. 2026 Apr 15;17:1757205. doi: 10.3389/fimmu.2026.1757205 (PMC13125040; doi:10.3389/fimmu.2026.1757205)

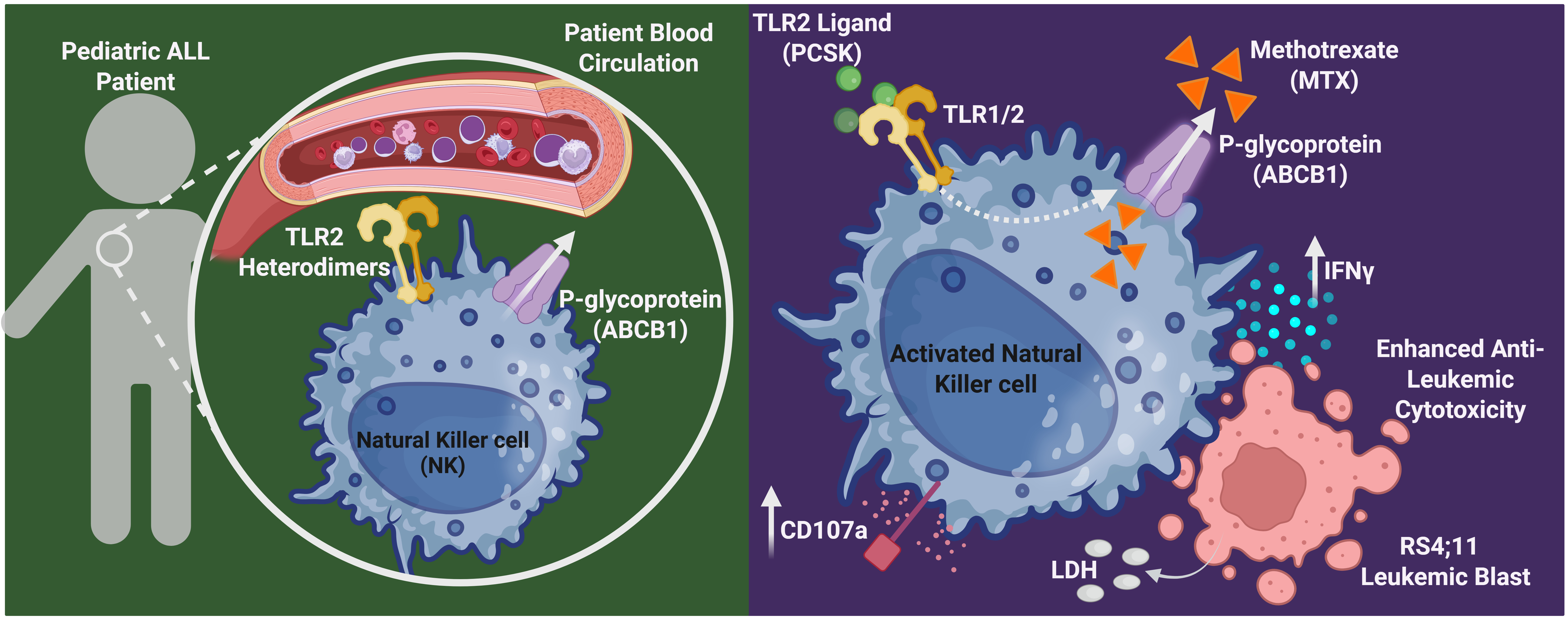

Supplement: Supplementary Figure 1 — The illustration depicts (Left) the high constitutive frequency of P-glycoprotein (P-gp) expression observed in NK cells from pediatric patients with acute lymphoblastic leukemia (ALL). (Right) it shows how TLR2 engagement triggers an intracellular signaling cascade (proposed mechanism) that optimizes P-gp efflux kinetics and boosts anti-leukemic effector functions, such as IFN-gamma secretion and CD107a degranulation, leading to enhanced cytotoxicity against leukemic blasts. This visual summary clarifies that TLR2 signaling acts as a functional booster to bridge the gap between basal protein expression and effective chemoresistance. [file Image1.jpeg]
